# 3,3′-Dimethyl-1,1′-[(1,3-dihy­droxy­propane-2,2-di­yl)dimethyl­idene]diimidazolium bis­(hexa­fluoro­phosphate)

**DOI:** 10.1107/S1600536810047677

**Published:** 2010-11-24

**Authors:** Ai-Lin Yuan, Chang-Sheng Wang, Ling-Hua Zhuang, Guo-Wei Wang

**Affiliations:** a Department of Light Chemical Engineering, College of Food Science and Light Industry, Nanjing University of Technology, Nanjing 210009, People’s Republic of China; b Department of Applied Chemistry, College of Science, Nanjing University of Technology, Nanjing 210009, People’s Republic of China

## Abstract

The title compound, C_13_H_22_N_4_O_2_^2+^·2PF_6_^−^, was prepared by the anion exchange of the dibromide ionic liquid with potassium hexa­fluoro­phosphate. The two imidazole rings are each planar (r.m.s. deviations = 0.0016 and 0.0060 Å) and make a dihedral angle of 45.3 (18)°. Intra­molecular O—H⋯F hydrogen bonds occur. Inter­molecular C—H⋯F, O—H⋯O and C—H⋯O hydrogen bonds stabilize the crystal structure.

## Related literature

For properties and applications of ionic liquids, see: Welton (1999 ▶). For dicationic ionic liquids, see: Liang *et al.* (2008 ▶); Geng *et al.* (2010 ▶). For the synthesis of the title compound, see: Cai *et al.* (2007 ▶); Cai & Liu, (2009 ▶). For bond-length data, see: Allen *et al.*, (1987 ▶).
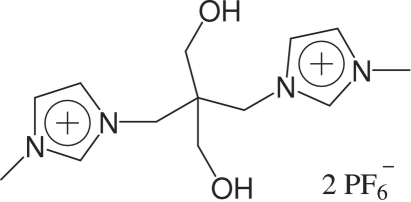


## Experimental

### 

#### Crystal data

C_13_H_22_N_4_O_2_^2+^·2PF_6_^−^
*M* *_r_* = 556.29
Orthorhombic, 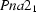

*a* = 14.622 (3) Å
*b* = 12.504 (3) Å
*c* = 12.165 (2) Å
*V* = 2224.2 (8) Å^3^
*Z* = 4
Mo *K*α radiation
μ = 0.31 mm^−1^
*T* = 295 K
0.20 × 0.10 × 0.10 mm

#### Data collection

Enraf–Nonius CAD-4 diffractometer
Absorption correction: ψ scan (North *et al.*, 1968 ▶) *T*_min_ = 0.940, *T*_max_ = 0.969
4082 measured reflections
2152 independent reflections
1861 reflections with *I* > 2σ(*I*)
*R*_int_ = 0.067
3 standard reflections every 200 reflections  intensity decay: 1%

#### Refinement

*R*[*F* ^2^ > 2σ(*F* ^2^)] = 0.066
*wR*(*F* ^2^) = 0.123
*S* = 1.00
4082 reflections
298 parameters
1 restraint
H-atom parameters constrained
Δρ_max_ = 0.20 e Å^−3^
Δρ_min_ = −0.17 e Å^−3^
Absolute structure: Flack (1983 ▶), 1930 Friedel pairs
Flack parameter: 0.01 (16)

### 

Data collection: *CAD-4* (Enraf–Nonius, 1989 ▶); cell refinement: *CAD-4*; data reduction: *XCAD4* (Harms & Wocadlo, 1995 ▶); program(s) used to solve structure: *SHELXS97* (Sheldrick, 2008 ▶); program(s) used to refine structure: *SHELXL97* (Sheldrick, 2008 ▶); molecular graphics: *SHELXTL* (Sheldrick, 2008 ▶); software used to prepare material for publication: *SHELXTL*.

## Supplementary Material

Crystal structure: contains datablocks global, I. DOI: 10.1107/S1600536810047677/rk2245sup1.cif

Structure factors: contains datablocks I. DOI: 10.1107/S1600536810047677/rk2245Isup2.hkl

Additional supplementary materials:  crystallographic information; 3D view; checkCIF report

## Figures and Tables

**Table 1:** Hydrogen-bond geometry (Å, °)

*D*—H⋯*A*	*D*—H	H⋯*A*	*D*⋯*A*	*D*—H⋯*A*
O1—H1*A*⋯F10	0.82	2.32	3.001 (8)	141
O2—H2*A*⋯O1^i^	0.82	1.97	2.787 (6)	175
C2—H2*B*⋯F7^i^	0.93	2.41	3.261 (11)	152
C13—H13*B*⋯O2^ii^	0.96	2.54	3.186 (7)	125

Symmetry codes: (i) 
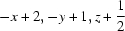
; (ii) 
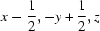
.

**Fig. 1.**
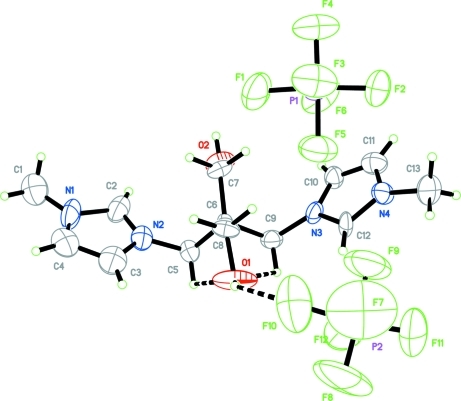
A view of the molecular structure of I showing the atom-numbering scheme. Displacement ellipsoids are drawn at 30% probability level. H atoms are presented as a small spheres of arbitrary radius. Dashed lines indicate hydrogen bonds.

**Fig. 2.**
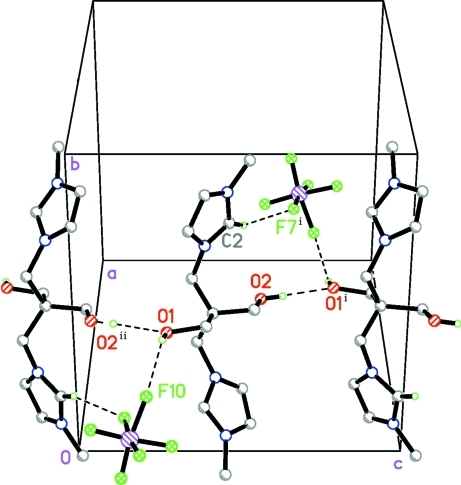
The crystal packing of I. Hydrogen bonds are drawn by dashed lines. Symmetry codes: (i) 2-x, 1-y, 1/2+z; (ii) -1/2+x, 1/2-y, z.

## supplementary crystallographic information

### Comment

Ionic liquids (*ILs*) have enjoyed considerable research interests in recent years because of their unique properties (Welton, 1999). Geminal dicationic *ILs* have been shown to possess superior physical properties in terms of thermal stability and volatility compared to traditional *ILs* (Liang *et al.*, 2008).

As part of our ongoing studies on new Geminal dicationic *ILs* (Geng *et al.*, 2010), we here report the crystal structure of the title compound I.

The atom-numbering scheme of I is shown in Fig. 1. There are exist intramolecular C—H···O and O—H···F hydrogen bonds, while intermolecular C—H···F, O—H···O and C—H···O hydrogen bonds stablize the crystal structure. All bond lengths are within normal ranges (Allen *et al.*, 1987). The imidazole ring is planar, with r.m.s. deviation 0.0016Å. The dihedral angles between two imidazole ring is 45.3 (18)°. There exist intermolecular C—H···F hydrogen bonds between hexafluorophosphate anions and imidazolium cations (Table 1, Fig. 1 and Fig. 2).

### Experimental

A mixture of 1-methylimidazole (2.05 g, 25 mmol) and 2,2-bis(bromomethyl)-propane-1,3-diol (2.60 g, 10 mmol) were stirred vigorously at 423 K for 8 h. After cooling to room temperature, the crude product was washed with acetonitrile. The resulting solid collected by filtration was treated with water (20 ml) as well as KPF_6_ (3.68 g, 20 mmol) and the reaction mixture was stirred at room temperature for 1 h. After filtration, the white solid was washed with ethanol and dried *in vacuo* to give the title compound I (5.02 g, 91%)(Cai *et al.*, 2007; Cai & Liu, 2009). M.p. 497–500 K. Crystals of I suitable for X-ray diffraction study were obtained by slow evaporation of methanol solution. ^1^H NMR (*DMSO*, δ, p.p.m.) 8.96 (s, 2 H), 7.72 (d, 2 H), 7.60 (d, 2 H), 5.29 (s, 2 H) 4.24 (s, 4 H), 3.87 (s, 6 H), 3.12 (s, 4 H).

### Refinement

In the both hexafluorophosphate groups, fluorine atoms have strong oscillations, while central P atoms are fixed. All H atoms were positioned geometrically, with C—H = 0.93Å, 0.96Å and 0.97Å for aromatic, methyl, methylene H, respectively. H atoms of hydroxy-groups were positioned geometrically too with O—H = 0.82Å. During refinement these H atoms were constrained to ride on their parent atoms, with *U*_iso_(H) = *xU*_eq_(C, O), where *x* = 1.2 for aromatic and methylene H atoms and *x* = 1.5 for methyl and hydroxyl H atoms.

### Crystal data

**Table:**

C_13_H_22_N_4_O_2_^2+^·2PF_6_^−^	*D*_x_ = 1.661 Mg m^−^^3^
*M**_r_* = 556.29	Melting point = 497–500 K
Orthorhombic, *P**n**a*2_1_	Mo *K*α radiation, λ = 0.71073 Å
Hall symbol: P 2c -2n	Cell parameters from 25 reflections
*a* = 14.622 (3) Å	θ = 9–13°
*b* = 12.504 (3) Å	µ = 0.31 mm^−^^1^
*c* = 12.165 (2) Å	*T* = 295 K
*V* = 2224.2 (8) Å^3^	Block, colourless
*Z* = 4	0.20 × 0.10 × 0.10 mm
*F*(000) = 1128	

### Data collection

**Table:**

Enraf–Nonius CAD-4 diffractometer	1861 reflections with *I* > 2σ(*I*)
Radiation source: fine-focus sealed tube	*R*_int_ = 0.067
graphite	θ_max_ = 25.4°, θ_min_ = 2.1°
ω/2θ–scans	*h* = 0→17
Absorption correction: ψ scan (North *et al.*, 1968)	*k* = 0→15
*T*_min_ = 0.940, *T*_max_ = 0.969	*l* = −14→14
4082 measured reflections	3 standard reflections every 200 reflections
2152 independent reflections	intensity decay: 1%

### Refinement

**Table:**

Refinement on *F*^2^	Hydrogen site location: inferred from neighbouring sites
Least-squares matrix: full	H-atom parameters constrained
*R*[*F*^2^ > 2σ(*F*^2^)] = 0.066	*w* = 1/[σ^2^(*F*_o_^2^) + (0.034*P*)^2^] where *P* = (*F*_o_^2^ + 2*F*_c_^2^)/3
*wR*(*F*^2^) = 0.123	(Δ/σ)_max_ < 0.001
*S* = 1.00	Δρ_max_ = 0.20 e Å^−^^3^
4082 reflections	Δρ_min_ = −0.17 e Å^−^^3^
298 parameters	Extinction correction: *SHELXL97* (Sheldrick, 2008)
1 restraint	Extinction coefficient: 0.105 (11)
Primary atom site location: structure-invariant direct methods	Absolute structure: Flack (1983), 1930 Friedel pairs
Secondary atom site location: difference Fourier map	Flack parameter: 0.01 (16)

### Special details

**Table:**

Geometry. All s.u.'s (except the s.u. in the dihedral angle between two l.s. planes) are estimated using the full covariance matrix. The cell s.u.'s are taken into account individually in the estimation of s.u.'s in distances, angles and torsion angles; correlations between s.u.'s in cell parameters are only used when they are defined by crystal symmetry. An approximate (isotropic) treatment of cell s.u.'s is used for estimating s.u.'s involving l.s. planes.
Refinement. Refinement of *F*^2^ against ALL reflections. The weighted *R*-factor *wR* and goodness of fit *S* are based on *F*^2^, conventional *R*-factors *R* are based on *F*, with *F* set to zero for negative *F*^2^. The threshold expression of *F*^2^ > σ(*F*^2^) is used only for calculating *R*-factors(gt) *etc*. and is not relevant to the choice of reflections for refinement. *R*-factors based on *F*^2^ are statistically about twice as large as those based on *F*, and *R*-factors based on ALL data will be even larger.

### Fractional atomic coordinates and isotropic or equivalent isotropic displacement parameters (Å^2^)

**Table:**

	*x*	*y*	*z*	*U*_iso_*/*U*_eq_	
O1	0.8641 (3)	0.5204 (5)	0.2865 (3)	0.119 (2)	
H1A	0.8104	0.5362	0.2756	0.179*	
N1	1.0848 (6)	0.8302 (5)	0.4610 (6)	0.103 (2)	
C1	1.1347 (5)	0.9027 (6)	0.5270 (7)	0.123 (3)	
H1B	1.1127	0.9740	0.5144	0.185*	
H1C	1.1983	0.8989	0.5082	0.185*	
H1D	1.1268	0.8845	0.6030	0.185*	
O2	1.0931 (3)	0.4708 (3)	0.5634 (3)	0.0826 (14)	
H2A	1.1025	0.4740	0.6298	0.124*	
N2	1.0515 (4)	0.6773 (4)	0.3942 (5)	0.0724 (16)	
C2	1.1092 (6)	0.7153 (6)	0.4672 (6)	0.092 (3)	
H2B	1.1525	0.6804	0.5101	0.111*	
N3	0.9585 (3)	0.2977 (4)	0.4254 (4)	0.0595 (13)	
C3	1.0013 (6)	0.7562 (7)	0.3474 (7)	0.101 (2)	
H3A	0.9577	0.7467	0.2927	0.121*	
N4	0.8598 (4)	0.1738 (4)	0.4570 (4)	0.0708 (15)	
C4	1.0258 (6)	0.8556 (7)	0.3952 (8)	0.098 (3)	
H4A	1.0027	0.9233	0.3801	0.117*	
C5	1.0500 (4)	0.5689 (4)	0.3557 (5)	0.0593 (15)	
H5A	1.1108	0.5385	0.3631	0.071*	
H5B	1.0342	0.5684	0.2783	0.071*	
C6	0.9823 (3)	0.4998 (4)	0.4183 (4)	0.0402 (12)	
C7	1.0003 (4)	0.4992 (5)	0.5411 (4)	0.0649 (16)	
H7A	0.9877	0.5695	0.5712	0.078*	
H7B	0.9598	0.4484	0.5765	0.078*	
C8	0.8832 (3)	0.5271 (5)	0.3995 (4)	0.0580 (15)	
H8A	0.8445	0.4778	0.4398	0.070*	
H8B	0.8709	0.5990	0.4257	0.070*	
C9	1.0043 (4)	0.3873 (4)	0.3704 (5)	0.0637 (15)	
H9A	0.9872	0.3866	0.2934	0.076*	
H9B	1.0698	0.3760	0.3744	0.076*	
C10	0.9922 (5)	0.2325 (5)	0.5067 (5)	0.0652 (19)	
H10A	1.0493	0.2387	0.5397	0.078*	
C11	0.9291 (5)	0.1587 (6)	0.5303 (6)	0.081 (2)	
H11A	0.9320	0.1074	0.5855	0.098*	
C12	0.8786 (4)	0.2570 (4)	0.3921 (5)	0.0599 (16)	
H12A	0.8428	0.2821	0.3344	0.072*	
C13	0.7815 (5)	0.1020 (6)	0.4552 (6)	0.109 (3)	
H13A	0.7397	0.1243	0.3988	0.164*	
H13B	0.7513	0.1040	0.5252	0.164*	
H13C	0.8019	0.0305	0.4404	0.164*	
P1	0.72571 (13)	0.35606 (18)	0.68464 (19)	0.0911 (6)	
F1	0.7669 (3)	0.4744 (3)	0.6875 (5)	0.1535 (18)	
F2	0.6868 (3)	0.2424 (3)	0.6720 (5)	0.1459 (18)	
F3	0.6246 (3)	0.4001 (4)	0.6840 (4)	0.1420 (16)	
F4	0.7290 (3)	0.3498 (5)	0.8136 (3)	0.151 (2)	
F5	0.7254 (4)	0.3651 (4)	0.5546 (4)	0.140 (2)	
F6	0.8266 (2)	0.3132 (4)	0.6825 (5)	0.1478 (18)	
P2	0.64385 (14)	0.33975 (18)	0.1878 (2)	0.0992 (7)	
F7	0.7457 (4)	0.3277 (4)	0.1659 (6)	0.179 (2)	
F8	0.6282 (7)	0.3840 (8)	0.0811 (7)	0.355 (7)	
F9	0.6598 (5)	0.3032 (5)	0.3039 (4)	0.220 (3)	
F10	0.6708 (5)	0.4574 (5)	0.2350 (7)	0.238 (4)	
F11	0.6079 (10)	0.2399 (7)	0.1625 (8)	0.390 (8)	
F12	0.5486 (5)	0.3672 (9)	0.2204 (9)	0.314 (6)	

### Atomic displacement parameters (Å^2^)

**Table:**

	*U*^11^	*U*^22^	*U*^33^	*U*^12^	*U*^13^	*U*^23^
O1	0.069 (3)	0.240 (6)	0.049 (2)	0.047 (4)	0.006 (2)	0.014 (3)
N1	0.109 (6)	0.068 (5)	0.131 (6)	−0.036 (5)	0.018 (5)	−0.028 (4)
C1	0.117 (7)	0.105 (6)	0.147 (8)	−0.006 (6)	0.008 (7)	0.006 (6)
O2	0.054 (3)	0.125 (4)	0.069 (3)	0.022 (3)	−0.011 (2)	−0.016 (3)
N2	0.065 (4)	0.067 (4)	0.086 (4)	−0.006 (3)	0.021 (3)	−0.005 (3)
C2	0.090 (6)	0.085 (6)	0.102 (6)	−0.023 (5)	0.021 (5)	−0.031 (5)
N3	0.063 (3)	0.050 (3)	0.066 (3)	−0.011 (3)	−0.019 (3)	−0.010 (3)
C3	0.085 (6)	0.110 (6)	0.108 (6)	−0.009 (6)	0.028 (5)	−0.005 (6)
N4	0.061 (3)	0.087 (4)	0.065 (3)	−0.029 (3)	−0.006 (3)	0.009 (3)
C4	0.077 (6)	0.100 (7)	0.116 (8)	0.008 (5)	0.038 (5)	0.012 (6)
C5	0.053 (3)	0.053 (3)	0.071 (4)	0.004 (3)	0.009 (3)	0.004 (3)
C6	0.047 (3)	0.036 (2)	0.037 (3)	−0.012 (3)	−0.006 (3)	0.005 (2)
C7	0.061 (4)	0.092 (4)	0.042 (3)	0.000 (3)	−0.003 (3)	−0.020 (3)
C8	0.047 (3)	0.087 (4)	0.041 (3)	0.021 (3)	0.004 (3)	0.015 (3)
C9	0.068 (4)	0.075 (4)	0.048 (3)	−0.007 (4)	0.005 (3)	−0.002 (3)
C10	0.071 (5)	0.052 (4)	0.072 (5)	−0.003 (4)	−0.016 (4)	0.005 (3)
C11	0.087 (5)	0.089 (5)	0.069 (5)	0.014 (5)	−0.003 (4)	0.004 (4)
C12	0.067 (4)	0.025 (2)	0.088 (4)	−0.004 (3)	−0.020 (4)	0.001 (3)
C13	0.077 (5)	0.133 (7)	0.118 (6)	−0.067 (5)	−0.007 (4)	−0.008 (5)
P1	0.0748 (13)	0.1251 (16)	0.0734 (12)	−0.0271 (12)	−0.0068 (14)	−0.0219 (14)
F1	0.166 (4)	0.136 (3)	0.159 (4)	−0.059 (3)	−0.010 (4)	−0.044 (4)
F2	0.170 (4)	0.120 (3)	0.148 (4)	−0.072 (3)	−0.012 (4)	−0.026 (4)
F3	0.077 (3)	0.234 (5)	0.116 (3)	−0.002 (3)	−0.004 (3)	0.021 (4)
F4	0.117 (4)	0.264 (7)	0.073 (3)	0.014 (4)	0.002 (3)	−0.007 (4)
F5	0.135 (5)	0.198 (6)	0.087 (4)	−0.029 (4)	0.016 (3)	0.012 (3)
F6	0.067 (3)	0.206 (5)	0.170 (4)	0.001 (3)	−0.014 (4)	−0.073 (4)
P2	0.0872 (16)	0.1174 (17)	0.0929 (15)	−0.0272 (13)	−0.0190 (16)	0.0416 (15)
F7	0.130 (5)	0.235 (6)	0.171 (5)	−0.026 (4)	0.038 (5)	−0.041 (6)
F8	0.375 (14)	0.489 (14)	0.201 (8)	−0.219 (11)	−0.182 (9)	0.238 (9)
F9	0.263 (8)	0.293 (8)	0.104 (5)	−0.080 (7)	−0.052 (5)	0.083 (5)
F10	0.206 (7)	0.153 (5)	0.355 (12)	−0.041 (5)	0.034 (7)	0.001 (7)
F11	0.70 (2)	0.233 (7)	0.241 (9)	−0.308 (11)	0.025 (13)	−0.029 (8)
F12	0.111 (5)	0.491 (15)	0.340 (14)	0.080 (7)	−0.002 (6)	0.009 (12)

### Geometric parameters (Å, °)

**Table:**

O1—C8	1.406 (6)	C6—C7	1.518 (7)
O1—H1A	0.8200	C6—C9	1.556 (8)
N1—C4	1.219 (9)	C7—H7A	0.9700
N1—C1	1.413 (9)	C7—H7B	0.9700
N1—C2	1.482 (10)	C8—H8A	0.9700
C1—H1B	0.9600	C8—H8B	0.9700
C1—H1C	0.9600	C9—H9A	0.9700
C1—H1D	0.9600	C9—H9B	0.9700
O2—C7	1.428 (7)	C10—C11	1.336 (8)
O2—H2A	0.8200	C10—H10A	0.9300
N2—C2	1.314 (8)	C11—H11A	0.9300
N2—C3	1.354 (9)	C12—H12A	0.9300
N2—C5	1.434 (6)	C13—H13A	0.9600
C2—H2B	0.9300	C13—H13B	0.9600
N3—C12	1.338 (6)	C13—H13C	0.9600
N3—C10	1.372 (7)	P1—F2	1.538 (4)
N3—C9	1.467 (7)	P1—F6	1.570 (4)
C3—C4	1.419 (11)	P1—F4	1.572 (4)
C3—H3A	0.9300	P1—F3	1.578 (4)
N4—C12	1.335 (7)	P1—F5	1.586 (5)
N4—C11	1.363 (8)	P1—F1	1.598 (4)
N4—C13	1.454 (7)	P2—F11	1.390 (6)
C4—H4A	0.9300	P2—F8	1.429 (6)
C5—C6	1.519 (6)	P2—F12	1.489 (7)
C5—H5A	0.9700	P2—F9	1.503 (5)
C5—H5B	0.9700	P2—F7	1.521 (6)
C6—C8	1.506 (6)	P2—F10	1.628 (6)
			
C8—O1—H1A	109.5	H8A—C8—H8B	108.3
C4—N1—C1	124.8 (9)	N3—C9—C6	115.2 (4)
C4—N1—C2	117.1 (8)	N3—C9—H9A	108.5
C1—N1—C2	118.0 (8)	C6—C9—H9A	108.5
N1—C1—H1B	109.5	N3—C9—H9B	108.5
N1—C1—H1C	109.5	C6—C9—H9B	108.5
H1B—C1—H1C	109.5	H9A—C9—H9B	107.5
N1—C1—H1D	109.5	C11—C10—N3	108.5 (6)
H1B—C1—H1D	109.5	C11—C10—H10A	125.8
H1C—C1—H1D	109.5	N3—C10—H10A	125.8
C7—O2—H2A	109.5	C10—C11—N4	106.1 (6)
C2—N2—C3	111.6 (7)	C10—C11—H11A	126.9
C2—N2—C5	124.9 (6)	N4—C11—H11A	126.9
C3—N2—C5	122.9 (7)	N4—C12—N3	107.3 (5)
N2—C2—N1	99.3 (7)	N4—C12—H12A	126.4
N2—C2—H2B	130.3	N3—C12—H12A	126.4
N1—C2—H2B	130.3	N4—C13—H13A	109.5
C12—N3—C10	107.8 (5)	N4—C13—H13B	109.5
C12—N3—C9	123.4 (5)	H13A—C13—H13B	109.5
C10—N3—C9	128.2 (5)	N4—C13—H13C	109.5
N2—C3—C4	109.2 (8)	H13A—C13—H13C	109.5
N2—C3—H3A	125.4	H13B—C13—H13C	109.5
C4—C3—H3A	125.4	F2—P1—F6	91.8 (3)
C12—N4—C11	109.9 (6)	F2—P1—F4	93.7 (3)
C12—N4—C13	129.4 (6)	F6—P1—F4	88.3 (3)
C11—N4—C13	120.7 (6)	F2—P1—F3	88.6 (3)
N1—C4—C3	102.7 (9)	F6—P1—F3	178.7 (4)
N1—C4—H4A	128.7	F4—P1—F3	92.9 (3)
C3—C4—H4A	128.7	F2—P1—F5	88.0 (3)
N2—C5—C6	112.6 (4)	F6—P1—F5	90.6 (3)
N2—C5—H5A	109.1	F4—P1—F5	178.0 (3)
C6—C5—H5A	109.1	F3—P1—F5	88.1 (3)
N2—C5—H5B	109.1	F2—P1—F1	175.5 (4)
C6—C5—H5B	109.1	F6—P1—F1	87.8 (3)
H5A—C5—H5B	107.8	F4—P1—F1	90.8 (3)
C8—C6—C7	108.5 (4)	F3—P1—F1	91.7 (3)
C8—C6—C5	114.9 (4)	F5—P1—F1	87.5 (3)
C7—C6—C5	112.5 (4)	F11—P2—F8	95.0 (6)
C8—C6—C9	110.3 (4)	F11—P2—F12	84.9 (6)
C7—C6—C9	109.2 (4)	F8—P2—F12	90.2 (6)
C5—C6—C9	101.1 (4)	F11—P2—F9	89.6 (5)
O2—C7—C6	110.6 (4)	F8—P2—F9	174.9 (6)
O2—C7—H7A	109.5	F12—P2—F9	88.0 (5)
C6—C7—H7A	109.5	F11—P2—F7	104.1 (7)
O2—C7—H7B	109.5	F8—P2—F7	92.1 (5)
C6—C7—H7B	109.5	F12—P2—F7	170.5 (5)
H7A—C7—H7B	108.1	F9—P2—F7	88.9 (4)
O1—C8—C6	109.0 (4)	F11—P2—F10	169.1 (6)
O1—C8—H8A	109.9	F8—P2—F10	90.5 (5)
C6—C8—H8A	109.9	F12—P2—F10	85.6 (5)
O1—C8—H8B	109.9	F9—P2—F10	84.6 (4)
C6—C8—H8B	109.9	F7—P2—F10	85.1 (3)
			
C3—N2—C2—N1	1.7 (7)	C7—C6—C8—O1	−175.8 (5)
C5—N2—C2—N1	173.0 (5)	C5—C6—C8—O1	57.2 (7)
C4—N1—C2—N2	−1.5 (9)	C9—C6—C8—O1	−56.3 (6)
C1—N1—C2—N2	−178.1 (6)	C12—N3—C9—C6	93.2 (6)
C2—N2—C3—C4	−1.6 (9)	C10—N3—C9—C6	−96.3 (7)
C5—N2—C3—C4	−173.1 (6)	C8—C6—C9—N3	−66.1 (6)
C1—N1—C4—C3	177.0 (7)	C7—C6—C9—N3	53.0 (6)
C2—N1—C4—C3	0.6 (10)	C5—C6—C9—N3	171.8 (5)
N2—C3—C4—N1	0.5 (9)	C12—N3—C10—C11	−6.0 (7)
C2—N2—C5—C6	95.0 (7)	C9—N3—C10—C11	−177.7 (6)
C3—N2—C5—C6	−94.7 (7)	N3—C10—C11—N4	4.7 (8)
N2—C5—C6—C8	68.1 (6)	C12—N4—C11—C10	−1.8 (8)
N2—C5—C6—C7	−56.8 (6)	C13—N4—C11—C10	176.3 (6)
N2—C5—C6—C9	−173.2 (5)	C11—N4—C12—N3	−1.8 (7)
C8—C6—C7—O2	178.5 (5)	C13—N4—C12—N3	−179.7 (6)
C5—C6—C7—O2	−53.2 (6)	C10—N3—C12—N4	4.7 (7)
C9—C6—C7—O2	58.2 (6)	C9—N3—C12—N4	176.9 (5)

### Hydrogen-bond geometry (Å, °)

**Table:**

*D*—H···*A*	*D*—H	H···*A*	*D*···*A*	*D*—H···*A*
C5—H5B···O1	0.97	2.56	2.910 (7)	101
C9—H9A···O1	0.97	2.46	2.831 (8)	102
O1—H1A···F10	0.82	2.32	3.001 (8)	141
O2—H2A···O1^i^	0.82	1.97	2.787 (6)	175
C2—H2B···F7^i^	0.93	2.41	3.261 (11)	152
C13—H13B···O2^ii^	0.96	2.54	3.186 (7)	125

Symmetry codes: (i) −*x*+2, −*y*+1, *z*+1/2; (ii) *x*−1/2, −*y*+1/2, *z*.
